# Scope and limitations of minimal invasive surgery in practice of pediatric surgical oncology

**DOI:** 10.4103/0971-5851.76198

**Published:** 2010

**Authors:** Sushmita Bhatnagar, Yogesh Kumar Sarin

**Affiliations:** *Department of Pediatric Surgery, B. J. Wadia Hospital for Children, Acharya Donde Marg, Parel, Mumbai – 400 018, India*; 1 *Maulana Azad Medical College and L. N. Hospital, New Delhi – 110 001, India*

**Keywords:** *Indications and limitations*, *minimally invasive surgery*, *solid tumors*

## Abstract

Management of Solid tumors in children needs a comprehensive multimodality protocol based treatment plan. Open surgical removal of the tumors occurring in any of the sites such as abdomen, thorax, chest wall, HFN (head, face, neck), brain and extremities, is the option which has been traditionally practiced even in the present era and in most of the centers. Nevertheless with the advances in science and technology and with ever increasing usage and expertise of laparoscopy in children, it’s application has extended to treatment of solid tumors in children. A review of the scope of such intervention as well as the limitations of minimal invasive surgery in this specialized field of pediatric surgery has been attempted in this article.

## INTRODUCTION

In 1911, the Swiss surgeon HC Jacobaeus reported the use of diagnostic laparoscopy in a large number of patients with a variety of conditions including cancer.[1] However, little progress was made in developing the role of minimal invasive surgery (MIS) in malignant disease for over 70 years. The technologic advances in the late 1980s with the introduction of miniaturized video cameras resulted in a dramatic escalation in the use of MIS. Its use in patients with malignancies has been slower to evolve; however, it is gaining greater acceptance for use in adult cancer patients. MIS is now commonly used for many applications in adult surgical oncology, including biopsy and resection of malignant disease in the chest and abdominal cavities, mediastinal and retroperitoneal lymph node dissection, staging of abdominal, pelvic and thoracic malignancies, and management of therapeutic complications.

As the general use of MIS in pediatric patients has lagged behind its use for adult patients, so has been its use in infants and children with malignancies. It has not, as yet, been widely accepted for more than diagnostic purposes and there are only a few reports describing the use of ablative MIS in infants and children with solid tumors. There are skeptics even today who feel that laparoscopy is not a complete form of laparotomy and that it might result in an inadequate or inappropriate management due to misdiagnosis.[2] The main concerns that have contributed to the limited use of minimal access surgery in these patients include the following.

Loss of tactile sensation: This is important when trying to evaluate the thoracic or peritoneal cavities for tumor spread and lymph node involvement when, for example, attempting therapeutic resection of pulmonary metastases in patients with osteosarcoma, or accurately staging a patient with an abdominal neuroblastoma.

Tumor spill: Spill of a Wilms’ tumor, for example, has a significant impact on tumor staging, therapeutic approach and, ultimately, prognosis. In addition, a number of pediatric tumors, such as pleuro-pulmonary blastoma or malignant thymoma, are not responsive to treatment modalities other than surgical resection. Tumor spill often leads to local recurrence that can be ultimately very difficult to treat.

Tumor recurrence at trocar sites: Although there are few reports of this phenomenon in adult patients, pediatric surgical oncologists continue to be concerned about this issue,[3] in spite of none of the reports encountering port site recurrence (PSR).[4]

Removal of tumor specimens from the abdominal or thoracic cavities: In order to retain the benefits of the minimal access approach, specimens are often morcellated so that they can be removed through the smaller incisions. Although it has been shown that the use of a tissue morcellator does not interfere with adequate histologic evaluation of the tissue,[5] concerns remain, especially with regard to the ability to assess the margins of resection and gross anatomic relationships.[6] Tumor size, margins and renal vein involvement cannot be assessed once Wilms’ tumor is morcellated.[7,8]

Nevertheless, as technology continues to advance and the skill and comfort of pediatric surgeons with minimally invasive approaches increase, its use for children with cancer is also likely to increase. The general benefits of a minimally invasive approach, such as cosmetic appearance, reduced hospital stay, faster recovery, no/minimal bowel adhesions, much less pain and discomfort, reduced analgesic requirement, minimal postoperative disability, shorter length of postoperative ileus and rapid wound healing are worth noting in these children with tumors who generally need long-term treatment.[9,10] A good example is the role of MIS in management of children with osteogenic sarcomas, who may undergo multiple procedures for resectable lung metastases.

## INDICATIONS AND ADVANTAGES OF MINIMAL INVASIVE SURGERY

Since 1971, when Klimkovich *et al*.[11] reported the use of thoracoscopy in children for diagnosis of mediastinal masses and cysts and masses of pulmonary parenchyma, there have been many reports from across the world, which have cemented the presence of MIS in surgical management of solid tumors in children.[12–21] The most recent expansion of MIS has been robotic surgery which provides an additional advantage in treating children with solid tumors of any age as described in a retrospective review of 100 consecutive cases.[22]

The various current indications and advantages of MIS in pediatric extra-cranial solid tumors are detailed below.

### Biopsy

Most of the extracranial, solid tumors of infancy and childhood are treated with a multimodal approach. The treatment paradigm for large tumors usually begins with an initial biopsy with subsequent delayed primary resection. Therefore, biopsy of a new mass in a child either by a laparoscopic or thoracoscopic approach is a common indication. These can often also be approached by a radiographically guided percutaneous biopsy, although direct visualization of the lesion often gives additional anatomic detail and may provide visual confirmation of adequate hemostasis. In addition, with the current increasing emphasis on the procurement of tumor tissue for biologic studies, the laparoscopic or thoracoscopic approach may be increasingly favored for its ability to obtain more tissue while remaining minimally invasive. The diagnostic accuracy for minimal access procedures has typically been high, generally being reported in the range of 85–100%. The avoidance of large incisions that often led to postoperative ileus and atelectasis, in conjunction with this high diagnostic accuracy permitting the prompt initiation of appropriate chemotherapy, is a significant advantage. In addition, diminished intraperitoneal and intrathoracic adhesions after a minimally invasive biopsy may be advantageous when performing second look or delayed primary surgery. An exception to this approach of biopsy of large tumors is Wilms’ tumor, in which transabdominal biopsy causes inevitable tumor spill into the peritoneal cavity thus upstaging the disease.

### Tumor excision

Tumors in the early stages can be excised by MIS, for example, neuroblastoma, Wilms’ tumor, etc. Several articles report laparoscopic excision of neuroblastoma, which claim superiority over conventional surgical procedures in early recovery and ability to begin chemotherapy soon after surgery. Though there are specific pre-requisites for laparoscopic excision of neuroblastoma, there is a clear and definite role of MIS in excising these tumors in children.[23–30] Nephrectomy for Wilms’ tumor is another common surgery performed in children. With preoperative chemotherapy, nephrectomy is possible using MIS and intraperitoneal morcellation of tumor may not be needed.[31] Several other tumors completely excised by laparoscopy reported in literature include adrenal tumors,[32–35] Altman tpe II–IV sacrococcygeal teratomas,[36] presacral ganglioneurofibroma[37] and ovarian teratomas (oophorectomy).[38–40] The resection of pheochromocytoma and even those arising from an extra-adrenal site laparoscopically[34] proves that pediatric surgical oncology MIS has advanced and is finding more and more indications of its use. Another breakthrough in MIS in pediatric oncology has been resection of liver tumors in children.[41–43] Very few reports are available, but the possibility of such a major surgery being done laparoscopically is exciting, provided the selection criteria for its use are met to ensure a successful outcome. MIS resection of osteoid osteoma of long bones is another landmark in the field of MIS in pediatric oncology, without bone grafting or internal fixation, excellent cosmetic appearance, early mobilization and no perioperative complications.[44]

### Staging

Although there have been significant improvements in the radiographic assessment of the extent of malignancy, discrepancies between radiographic and surgical staging can occur. With the development and acceptance of MIS may come a greater emphasis on the need for surgical/pathologic staging. Accurate staging will become even more important as pediatric protocols attempt to decrease the intensity of treatment, while maintaining high rates of cure currently achieved for many pediatric malignancies. Lymph nodes in the chest and abdomen can be easily sampled through a minimally invasive approach to stage thoracic and abdominal tumors such as neuroblastoma, germ cell tumors, etc. Occasionally, intra-abdominal lymph nodes may need to be evaluated for extra-abdominal primaries also. For example, iliac lymph nodes need to be evaluated in patients with testicular tumors when an abnormality is detected on computed tomography (CT) scan. This is important for staging in rhabdomyosarcoma and can, additionally, be therapeutic for patients with a germ cell tumor of the testis.

During staging, the liver surface can be inspected for small, metastatic deposits and biopsy performed under direct vision, with the confirmation that hemostasis has been achieved. The peritoneal and pleural surfaces, sites of tumor spread not always accurately assessed by imaging studies, can be completely evaluated for disease. Finally, nodules of the pulmonary parenchyma, detected by CT as part of a staging evaluation, can be biopsied to confirm or exclude the presence of metastatic disease. This determination can have a significant impact on the treatment plan and ultimate prognosis for children with cancer.

Radiographic imaging has shown an error in staging when compared to surgical staging of approximately 30% for children with Hodgkin’s disease. However, because treatment of these patients has been so successful, with a high cure rate and low morbidity, surgical staging is no longer a part of the routine staging of these children. However, there is still interest in an MIS approach to the evaluation of nodal regions with equivocal CT in cases where disease stage and, consequently, treatment will be significantly impacted. This is particularly true when distinguishing stage II from stage III Hodgkin’s disease where the use of alkylating agents and radiation therapy is being considered. Whether routine, complete surgical staging will return to favor as the minimal morbidity and diagnostic accuracy of laparoscopy in evaluating the liver, spleen and lymph nodes is recognized, is uncertain.

### Determination of resectability

The determination of resectability of a primary tumor is a very useful indication for laparoscopy or thoracoscopy. MIS has been often used for the evaluation of liver tumors, tumors of the chest wall and large pelvic tumors, especially ovarian tumors. The minimally invasive approach can be used to evaluate anatomic relationships, invasion of vital structures and to assess whether multifocal disease is present. If a tumor is determined to be unresectable, a biopsy can be easily performed at the same time if this has not already been done.

### Monitoring response to neo-adjuvant chemotherapy

During the multimodality treatment of solid tumors, one needs to monitor the response of solid tumor to neo-adjuvant chemotherapy. Imaging techniques frequently could provide only limited information. Laparoscopy can be thence used to visualize the tumor and help in formulating further management plan, for example, neuroblastomas, germ cell tumor, etc. Also, laparoscopy could be useful for excising the residual mass in the same sitting. Liver biopsy could be safely done during assessment via laparoscope when indicated.

### Second-look, recurrence, metastatic disease

Second-look operations can be performed even after a primary open resection. Tumor recurrence, both locoregional and metastatic, can be documented through a minimal access approach. This has been done most often for pulmonary metastases and retroperitoneal sites. Because pulmonary metastases are usually peripheral lesions, they can be approached easily by thoracoscopy. In certain histologic types, resection of metastatic lesions may be both diagnostic and therapeutic, favorably influencing long-term survival. One drawback, however, to the thoracoscopic approach is the inability to palpate the lung to exclude the presence of smaller metastatic foci. It is unclear at this time, however, whether failing to identify and resect these lesions early will impact on patient survival.

### Infectious and other treatment associated complications

Pediatric cancer patients frequently undergo intensive, multimodal therapy and a number of complications that require surgical intervention can arise during the course of their treatment. Many of these can be dealt with using a minimally invasive approach. Patients will often become anorexic as a result of their treatment and may benefit from a laparoscopic gastrostomy tube placement. Children who are unable to tolerate enteral feeds of any type and require total parenteral nutrition (TPN) occasionally develop cholelithiasis as a consequence. Laparoscopic cholecystectomy can be performed in these patients for symptomatic stones or for acute cholecystitis. Patients with brain tumors may develop symptomatic gastroesophageal reflux. If the reflux is refractory to medical management, they may benefit from a laparoscopic Nissen fundoplication. Laparoscopic oophoropexy is often performed for females who are to undergo abdominopelvic irradiation.

The children with malignancies are immunosuppressed both because of their malignancy as well as their treatment. MIS can be a very useful technique for evaluating lesions seen on radiographic work-up. Diagnostic tissue can be obtained to distinguish among tumor, a benign process and an infectious process and tissue for culture obtained to identify particular inciting organisms. This situation arises most frequently with lesions (or diffuse processes) of the lung, in which a thoracoscopic lung biopsy can be performed, but may occur in the liver or retroperitoneum.

### Ligation of tumor vessels

Laparoscopic ligation of vessels before surgical intervention is of definite benefit in terms of ease of surgery and bloodless field intraoperatively. Sacrococcygeal teratoma is the only tumor wherein laparoscopic ligation of median sacral artery has been successfully performed by several authors.[45–47]

## CONTRAINDICATIONS OF MINIMAL INVASIVE SURGERY

Absolute contraindications to MIS in pediatric surgical oncology include:

- associated coagulation disorder,
- respiratory compromise of any etiology, and
- an infective focus, especially in the anterior abdominal wall, especially in the periumbilical region.

Relativecontraindications include:

- extensive previous surgery that has resulted in dense intra-abdominal/ thoracic adhesions and
- ablative MIS when tumor is very huge as it may lead to tumor spillage with its sequelae.

## COMPLICATIONS OF MINIMAL INVASIVE SURGERY

The conversion rate mentioned for pediatric surgical MIS procedures is usually quoted between 7 and 10%,[48] but they are understandably higher in diagnostic and ablative procedures of tumors (29%).[18] Judicious conversion to open surgery is not to be considered as a complication in any case. However, several complications of MIS have been cited in the literature.

### Atelectasis

It is the most dreaded complication of any MIS procedure; this may hamper postoperative recovery.[15]

### Trocar site herniation

Trocar site herniation is another known complication of MIS in children[49] and is seen to occur more often in children less than 5 years of age.[50]

### PSR

The complication specific to cancer surgery in MIS is that of PSR. PSR is defined as local, circumscribed tumor growth at the site of one or more trocar sites or at the incision site after laparoscopic or thoracoscopic surgery for cancer. PSR is localized within the abdominal or thoracic walls, within the scar tissue and involves initially the dermis and the subcutaneous fat and usually not the muscular layer. If these lesions occur within a few months (approximately up to 200 days) after endoscopic surgery, they fit into the criteria of PSR. PSR is not identical with peritoneal metastases, serosal invasion, skin metastases. A comprehensive literature search revealed only one case of PSR reported in pediatric age group after thoracoscopic resection of pulmonary metastases owing to osteogenic sarcoma.[51] On the contrary, a study conducted by the Japanese Society of Pediatric Endosurgeons did not reveal any PSR amongst 29 institutes which performed MIS procedures in children with tumors, and thus concluded that it is a rare phenomenon in children.[3]

### Tumor spillage after morcellation

This is the most dreaded problem during extraction of the tumor. A studyon cytologic washings retrieved from the bag used for specimen collection and morcellation revealed that malignant cells can be identified from the washing of the bag which had contained morcellated tissue, thus implying that whenever there is tumor spillage during morcellation, malignant cells do get lodged into surrounding tissue and alter the stage of the tumor.[52] Pathologic examination of the morcellated tissue is definitely more challenging, tedious and time consuming. As of today, majority would recommend separate incision wherein the tumor is extracted *in toto* without spillage.

## OTHER UNKNOWN FEARS

MIS is still evolving. A lot of questions still remain unanswered:

1. whether tumor recurrence rates are higher with MIS,
2. whether trocar site recurrence and seedling occurs with MIS,
3. whether incomplete resections with MIS have different implications as compared to open surgery and
4. Is there dissemination of tumor cells after carbon dioxide insufflation?

## CONCLUSIONS

The role of MIS in all children with tumors is still debatable. MIS is not simply utilization of new technology, but rather a philosophy that aims to minimize the physiologic consequences of an operation, which include pain, scarring, stress response and disability. In comparison to open surgery, MIS has several advantages: less pain, faster recovery time and improved cosmesis (much smaller incisions). However, any surgical technique to be established as better than the standard requires a long-term dedicated study about its applicability as well as complications and safety of use. Clearly, carefully controlled randomized studies are needed to help determine the benefits and drawbacks of this new, evolving methodology.

In the recent times, with improved instrumentation and skills in pediatric MIS, the treatment and staging of tumors in children laparoscopically is becoming more and more popular. There are still several limitations to the use of laparoscopy in pediatric oncology and it is doubtful that MIS will replace open surgery but future technical developments and its applications are difficult to predict.
